# Bilateral Anterior Ischaemic Optic Neuropathy as First Manifestation of Henoch-Schönlein Purpura (IgA Vasculitis)

**DOI:** 10.31138/mjr.32.1.81

**Published:** 2020-10-19

**Authors:** Antonios Ragkousis, Tina Xirou, Georgios Bontzos, Evangelos Gkoumas, Evgenia Kontou

**Affiliations:** Department of Ophthalmology, “Korgialenio-Benakio” Red Cross Hospital, Athens, Greece

**Keywords:** Henoch-Schönlein purpura, IgA vasculitis, anterior ischaemic neuropathy, macular star

## Abstract

A 46-year-old man was referred to our department complaining of a bilateral progressive decrease in his visual acuity. Fundus examination revealed bilateral optic disc oedema, indicative of anterior ischaemic neuropathy (AION), and a macular star in the right eye. Laboratory analysis showed low haematocrit and haemoglobin, elevated creatinine, and increased erythrocyte segmentation rate and C-reactive protein level. Physical examination revealed the presence of purpuric rash on the trunk and the extremities. During the investigation we performed a complete laboratory and imaging examination for autoimmune collagen diseases, vasculitides and infectious diseases, which were all negative. Histologic findings of renal biopsy were compatible with IgA glomerulonephritis and thus Henoch-Schönlein purpura (HSP) diagnosis was established. The patient was treated with methylprednisolone and cyclophosphamide. Six months later, his renal function and his visual acuity had improved, and the rash had subsided. This is a rare case of AION in a patient with HSP.

## INTRODUCTION

Henoch-Schönlein purpura (HSP), also known as IgA vasculitis, is a small vessel vasculitis, more commonly seen in paediatric population. Histopathologically, it is characterized by IgA deposits in vessel walls. It predominantly affects the skin, the gastrointestinal tract, the kidneys, and the joints. Clinical manifestations include palpable erythematous lesions mostly over the lower extremities and buttocks, bowel angina, arthralgia/arthritis, and microscopic haematuria. In adults, as aforementioned, HSP is much less frequent; however, when it occurs, the renal involvement is more severe and can lead to renal failure due to rapidly progressive glomerulonephritis. IgA nephropathy (IgAN) is a limited non-systemic renal disease that bears identical histopathological abnormalities. The two clinical entities are considered to be part of the same disease spectrum, as both can be encountered consecutively in the same patient.^1^

Ophthalmic manifestations of HSP and IgAN are rare. HSP is most often associated with anterior uveitis,^2^ whereas IgAN is usually linked to scleritis and epicleritis.^3^ Other ocular findings include sclerokeratitis, angle-closure glaucoma secondary to ciliochoroidal effusion, Vogt-Koyanagi-Harada syndrome, serous retinal detachments, posterior scleritis and perifoveal drusenoid deposits for IgAN, while regarding HSP: central retinal artery occlusion, central retinal vein occlusion, cystoid macular oedema, multiple retrohyaloid haemorrhages, subperiosteal orbital haematomas, and combined peri-orbital, facial and scalp oedema.^4,5^ There are only few references about posterior segment involvement with retinal vasculitis.^6^ One case of anterior ischaemic optic neuropathy (AION) secondary to HSP has been reported.^7^ Here, we present a case of a patient with HSP with bilateral AION accompanied by the presence of a macular star. This was the patient’s first clinical manifestation of the disease.

## CASE DESCRIPTION

**Figure 1.**
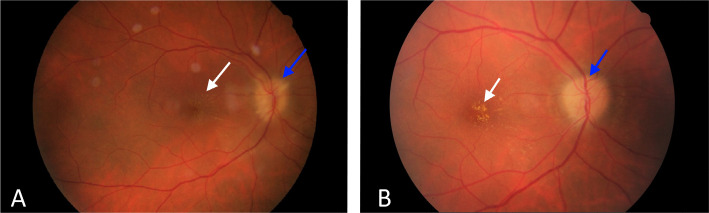
**A.** Colour fundus photography of the right eye showing an oedematous pale “chalky white” optic disc (blue arrow) and a macular star (white arrow); **B.** Colour fundus photography of the right eye, 4 months later, demonstrating a white atrophic optic disc (blue arrow) and some remaining macular hard exudates (white arrow).

**Figure 2.**
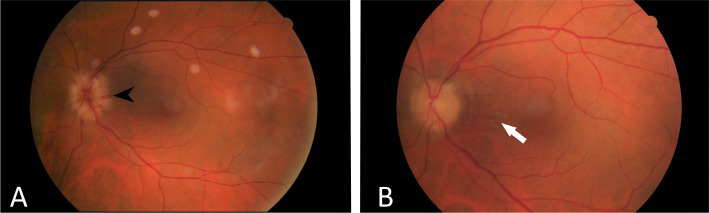
**A.** Colour fundus photography of the left eye showing an oedematous optic disc with flame-shaped haemorrhages (black arrowhead); **B.** Colour fundus photography of the left eye, 4 months later, showing an atrophic optic disc and some macular hard exudates (white arrow).

**Figure 3.**
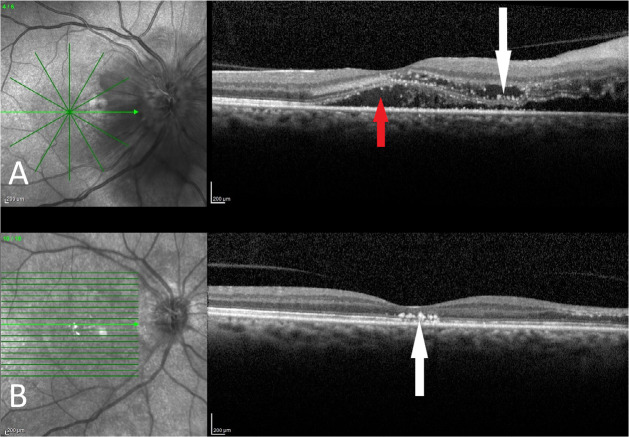
**A.** OCT of the right eye showing subretinal fluid (red arrow) extending from the optic disc to the macula and accompanying hard exudates (white arrow); **B.** OCT of the right eye, 4 months later. The subretinal fluid has disappeared but there are some residual hard exudates (white arrow) at the fovea

**Figure 4.**
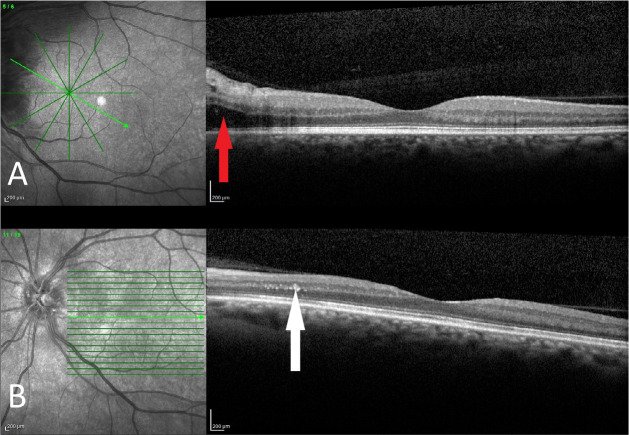
**A.** OCT of the left eye showing subretinal fluid nasal to the fovea (red arrow); **B.** OCT of the left eye, 4 months later. The subretinal fluid has disappeared but there are some hard exudates (white arrow) in the papillomacular region.

A 46-year-old male was referred to our department complaining of a 10-day painless progressive decrease in visual acuity of his right eye with a similar course of 3 days on his left eye. There was no history or symptoms suggestive of preceding upper respiratory tract infection. Apart from having glucose-6-phosphatase dehydrogenase (G6PD) deficiency, the patient had an otherwise unremarkable past medical history. He was receiving no medication and had no smoking history. Ophthalmic examination revealed best corrected visual acuity (BCVA) of hand motion in the right eye, and 4/10 in the left eye. Ocular motility was normal without associated pain or diplopia. Pupil examination revealed a relative afferent pupillary defect (RAPD) in the right eye, and Ishihara colour testing demonstrated a defect in colour vision. Confrontational visual field testing showed diffuse field constriction bilaterally. Slit lamp examination of the anterior segment was unremarkable. Intraocular pressures (measured by Golmann applanation tonometry) was 14 mmHg in both eyes. Dilated fundus examination unveiled a pale “chalky white” oedematous optic disc bilaterally, flame – shaped haemorrhages around the optic disc of the left eye and hard exudates that were forming a macular star in the right eye (**Figures 1A** and **2A**). Optical Coherence Tomography (OCT) of the right eye showed the presence of subretinal fluid that was extending from the oedematous optic disc to the macula, as well as the presence of hard exudates (**Figure 3A**). Similar findings were observed in the left eye, however there was less subretinal fluid (**Figure 4A**).

After the ophthalmological examination was completed, the most likely diagnoses were infectious neuroretinitis, because of the macular star and arteritic anterior ischaemic neuropathy (A-AION) because of the image of the oedematous optic discs. Although most cases of neuroretinitis are unilateral, bilateral cases have also been reported. The majority of A-AION cases are known to be caused by giant cell arteritis (GCA). However, our patient was very young (GCA is very rare under the age of 50), so we could not determine a presumable cause for A-AION.

**Figure 5.**
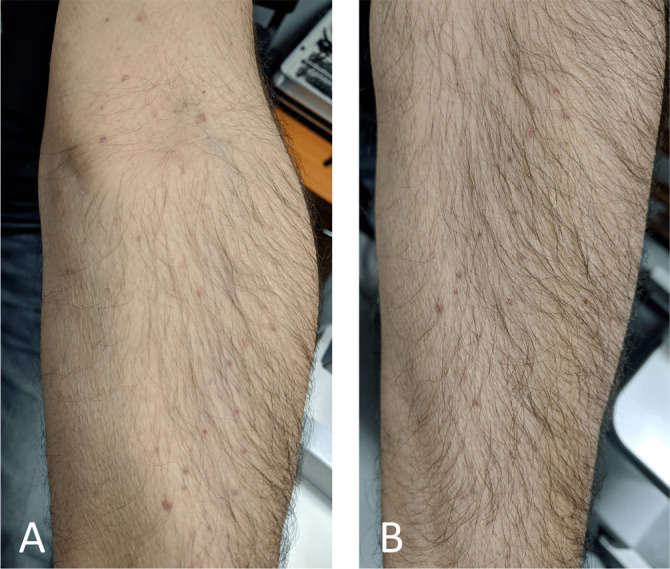
Purpuric rash. **A.** Over the flexor surfaces of the upper extremities; **B.** Over the extensor surfaces of upper extremities.

On physical examination, a purpuric rash was observed over the extremities and the trunk (**Figure 5**). The patient was afebrile and there were no arthralgias, abdominal pain, headache, malaise, nights sweats or reported weight loss. No oral aphthous or genital ulcers were found. His arterial blood pressure was 140/90. Regarding blood testing, full blood count revealed a low haematocrit (22,1%), and haemoglobin (7,4 g/dL), with normal white blood cells (10,5 x 10^3^ /μL) and a moderately elevated platelet count (498 x 10^3^ /μL). The patient also had an increased erythrocyte sedimentation rate (140mm/hr) and an elevated C-reactive protein level (64,80 mg/L). Biochemistry demonstrated a creatinine value of 4,4 mg/dL and blood urea nitrogen level of 59 mg/ dL. Coagulation tests showed increased fibrinogen (655 mg/dL, with reference range 200–400 mg/dL), increased d-dimers (2,43 μg/mL, with reference range <0.5 μg/mL) and a prothrombin time international normalized rate (PT - INR) of 1.13. Furthermore, imaging investigations such as chest x-ray, abdominal and renal ultrasound and brain computed tomography were unremarkable.

Based on the above findings, the decision was made to admit the patient to the Nephrology department for treatment and further investigation of his azotaemia. Microscopic haematuria (10–15 red blood cells/high power field) was noted on urinalysis, and 24-hour urine protein test revealed proteinuria (1,92 g/24h).

Subsequently, a further antibody screening was carried out; amongst the tests that were included were: Antinuclear antibody (ANA), anti-dsDNA, anti-cardiolipin, anti-PR3, anti-MPO, Anti-GBM antibodies, cryoglobulins, and Ra test. C3, C4 complement, lupus anti-coagulant-sensitive aPTT and serum angiotensin converting enzyme (SACE) were normal. Total serum protein was elevated (86 g/L) with globulin fraction increase (albumin = 44 g/L, globulin = 42 g/L). Serum protein electrophoresis revealed polyclonal gamma-globulin increase and alpha-1 globulin increase. Due to the presence of the macular star an infectious diseases screening was carried out to exclude the infectious causes of neuroretinitis. Treponema pallidum haemagglutination (TPHA), rapid plasma reagin (RPR) tests for syphilis and antibodies for Bartonella Henselae, Borrelia Burgdorferi, Leptospira, Toxoplasma gondii were all negative. Serology for HBV, HCV and HIV was also negative. Notably, the renal biopsy demonstrated mesangial proliferation and glomerular crescent formation within the Bowman’s space - the latter being indicative of a rapidly progressive glomerulonephritis. Direct immunofluorescence of renal biopsy demonstrated granular deposits of IgA in the mesangium, suggestive of HSP.

Immediately after the first laboratory results and after the renal biopsy was collected, the patient was commenced on high dose intravenous methylprednisolone at 1g/day for the first 3 days. Treatment was then continued with an oral methylprednisolone regimen at an initial dose of 1mg/kg/day, followed by a 6-month tapering period. This was given in combination with pulsed intravenous cyclophosphamide at a dose of 0,5 g/m^2^ BSA monthly for 6 months. The patient subsequently responded well to the treatment and his renal function improved. Furthermore, he noticed a significant improvement in visual acuity regarding his left eye, which 3 days later had reached 7/10; his right eye remained at hand motion. Due to the presence of nephropathy, it was deemed inappropriate to perform fluorescein angiography at that stage.

Four months later, the renal function of the patient was stable; his creatinine was 2.1 mg/dL and the estimated glomerular filtration rate (Cockcroft-Gault formula) was 50 mL/min. Patient’s BCVA had improved to counting fingers at 1 ft in the right eye, and 10/10 in his left eye. The presence of macular oedema (subretinal fluid) was an aggravating factor for the reduction of the visual acuity in addition to the optic disc oedema. Therefore, visual acuity was improved, owing to both optic discs’ swelling recession and macular oedema recession. Despite this significant improvement in visual acuity, his visual field remained critically compromised in both eyes, as shown in Humphrey automated perimetry. Fundoscopic examination revealed secondary optic disc atrophy bilaterally. There were also some remaining hard exudates (**Figures 1B** and **2B**). OCT scans demonstrated resolution of the subretinal fluid in both eyes (**Figures 3B** and **4B**).

## DISCUSSION

Anterior ischaemic optic neuropathy (AION) is divided into non-arteritic (NA-AION) and arteritic (A-AION). NA-AION is the most common form of ischaemic optic neuropathy. It typically occurs in patients older than 50 years with cardiovascular risk factors such as diabetes mellitus, hypertension, hyperlipidaemia, obstructive sleep apnoea, and nocturnal arterial hypotension. Another predisposing factor is a crowded optic disc with small cup-to-disc ratio. NA-AION is manifested as sudden, isolated, painless monocular vision loss with optic disc swelling.^8^

A-AION is caused by occlusion secondary to inflammation of the short posterior ciliary arteries due to immune-mediated vasculitis. GCA is the primary aetiology, although rarely other types of vasculitis can also cause it, eg, polyarteritis nodosa, microscopic polyangiitis, systemic lupus erythematosus, Wegener’s granulomatosis, Churg-Strauss syndrome, and HSP.^9^ The clinical presentation of A-AION is similar with that of NA-AION. The degree of visual loss is more profound in A-AION. Amaurosis fugax and transient diplopia are ominous signs of impending visual loss. The affected oedematous optic disc is characteristically pale “chalky white” immediately in A-AION, whereas pallor is delayed in NA-AION. The longer the time interval the A-AION stays untreated without steroids, the higher the risk of second eye involvement in days to weeks.

In our case, the clinical findings of bilateral optic disc pallor, in combination with a 7-day interval between the onset of visual symptoms in right - and subsequently left eye - pointed towards the probable diagnosis of A-AION. This was further supported by the patient’s rapid corticosteroid response. In addition, the patient’s young age, as well as his lack of symptoms of scalp tenderness, headache and jaw claudication, minimized the chance of GCA of being possible cause for his presentation.

It should be stated that the patient fulfilled both the American College of Rheumatology diagnostic criteria for Henoch-Schönlein purpura and the most recent European League Against Rheumatism/Paediatric Rheumatology International Trials Organization/Paediatric Rheumatology European Society (EULAR/PRINTO/RES) criteria.^1^ The latter include non-thrombocytopenic purpura (commonly palpable and in crops) or petechiae, as a mandatory criterion, plus at least one the following :

1) Diffuse abdominal pain;
2) Typically leucocytoclastic vasculitis with predominant IgA deposit or proliferative glomerulonephritis with predominant IgA deposit, in histopathology;
3) Arthritis or arthralgias;
4) Renal involvement with proteinuria >0.3 g/24 h or >30 mmol/mg of urine albumin/creatinine ratio on a spot morning sample, haematuria or red blood cell casts: >5 red blood cells/high power field or red blood cells casts in the urinary sediment or ≥2+ on dipstick.

Our patient fulfilled the mandatory criterion of the purpura, and also criteria number 2 and 4.

An interesting finding in this case was the macular star. The macular star consists of hard exudates in Henle’s layer that are arranged in a stellate pattern around the fovea. These lipid-rich exudates leak from the capillaries of the disc due to a defect in vessel’s wall blood-tissue barrier and extend into the papillomacular region. Non-infectious causes are uncommon but various inflammatory conditions can mimic neuroretinitis. It has been reported as part of vasculitis like Behcet’s disease and polyarteritis nodosa.^10^ In addition, Wolfenberger TJ et al.^6^ reported the presence of a foveal hard exudate accumulation in a patient with retinal vasculitis and IgA nephropathy. A reasonable explanation would be the excessive, long-term blood vessel inflammation in vasculitis that leads to increased leakage due to vascular perfusion and permeability changes. A hypertensive spike due to chronic kidney disease and nephrotic syndrome could further aggravate this mechanism.

In summary, when an arteritic anterior ischaemic optic neuropathy, which is not characteristic of a GCA, is detected, other systemic diseases should be considered as part of a broader differential diagnosis. Coexisting clinical features should be carefully evaluated. In cases of a skin rash or glomerulonephritis, histopathological examination including direct immunofluorescence should be carried out in order to establish the diagnosis. In certain instances (such as the one described above) prompt treatment with corticosteroids would improve the prognosis and prevent further involvement of the fellow eye.

Although HSP is rare in adults, it is the most common childhood vasculitis, and it can present with a variety of systemic symptoms and signs. On the rare occasion, the disease might manifest primarily via the eyes, preceding the appearance of other systemic symptoms. Therefore, clinicians should report and record all possible ocular, and non-ocular manifestations and should be aware of them.

## CONSENT

Written informed consent for the publication of the case was obtained from the patient.
